# Efficacy of nasal irrigation and oral rinse with sodium bicarbonate solution on virus clearance for COVID-19 patients

**DOI:** 10.3389/fpubh.2023.1145669

**Published:** 2023-03-15

**Authors:** Tairong Wang, Yue Zhang, Rong Zhang, Ye Mao, Junhai Yan, Yiwen Long, Qiaofeng Chen, Xiaojing Li, Huixiang Wang, Shuai Huang, Chao Zhu, Bin Teng, Xu Wang

**Affiliations:** ^1^Department of Health Management Branch, Ruijin Hospital Luwan Branch, Shanghai Jiao Tong University School of Medicine, Shanghai, China; ^2^Department of Pathology, Ruijin Hospital and College of Basic Medical Sciences, Shanghai Jiao Tong University School of Medicine, Shanghai, China; ^3^Department of Respiratory, Ruijin Hospital Luwan Branch, Shanghai Jiao Tong University School of Medicine, Shanghai, China; ^4^Intensive Care Unit, Ruijin Hospital Luwan Branch, Shanghai Jiao Tong University School of Medicine, Shanghai, China; ^5^Department of General Surgery, Ruijin Hospital Luwan Branch, Shanghai Jiao Tong University School of Medicine, Shanghai, China; ^6^Nursing Department, Ruijin Hospital Luwan Branch, Shanghai Jiao Tong University School of Medicine, Shanghai, China; ^7^Department of Information Technology, Ruijin Hospital Luwan Branch, Shanghai Jiao Tong University School of Medicine, Shanghai, China; ^8^General Department, Wuliqiao Community Health Service Center, Shanghai, China; ^9^Nanning Jiuzhouyuan Biotechnology Co. Ltd., Nanning, China; ^10^Key Laboratory of Cell Differentiation and Apoptosis of Chinese Ministry of Education, Shanghai Jiao Tong University School of Medicine, Shanghai, China

**Keywords:** nasal irrigation, oral rinse, virus clearance, sodium bicarbonate solution, COVID-19

## Abstract

**Background:**

Recent studies have shown that the infectivity of severe acute respiratory syndrome coronavirus 2 (SARS-CoV-2) is reduced under alkaline conditions. The purpose of this study is to assess the effect of nasal irrigation and oral rinse with sodium bicarbonate solution on virus clearance among COVID-19 patients.

**Materials and methods:**

COVID-19 patients were recruited and randomly divided into two group, i.e., the experimental group and the control group. The experimental group received regular care plus nasal irrigation and oral rinse with 5% sodium bicarbonate solution, while the control group only received regular care. Nasopharyngeal and oropharyngeal swab samples were collected daily for reverse transcription-polymerase chain reaction (RT-PCR) assays. The negative conversion time and hospitalization time of the patients were recorded, and the results were statistically analyzed.

**Results:**

A total of 55 COVID-19 patients with mild or moderate symptoms were included in our study. There was no significant difference in gender, age and health status between the two groups. The average negative conversion time was 1.63 days after treatment with sodium bicarbonate, and the average hospitalization time of the control group and the experimental group were 12.53 and 7.7 days, respectively.

**Conclusions:**

Nasal irrigation and oral rinse with 5% sodium bicarbonate solution is effective in virus clearance for COVID-19 patients.

## 1. Introduction

The emergence and spread of severe acute respiratory syndrome coronavirus 2 (SARS-CoV-2) has led to the global pandemic of coronavirus disease 2019 (COVID-19) (1). As of October 31, 2022, there have been more than 620 million confirmed cases of SARS infection worldwide, with more than 6.5 million cumulative deaths (2). The Omicron variant of SARS-CoV-2 has become the main epidemic strain owing to its increased infectivity (3), which led to the outbreak in Shanghai in March 2022. The majority of infected patients in Shanghai showed no symptoms or only mild to moderate symptoms. Only very few patients were severely ill, and most of those who died had serious underlying diseases. Although the mortality rate is extremely low, the coronavirus is highly contagious (4), resulting in the shutdown of Shanghai for more than 2 months. Thus, it is essential and urgent to slow down the spread of SARS-CoV-2.

It has been demonstrated that nasal irrigation and oral rinse with physiological saline solution is a simple and effective way to reduce acute infection of the nasal and oral pharyngeal mucosa (5, 6). This is confirmed by numerous clinical studies that nasal irrigation with saline (0.9%) and hypertonic saline (2.3%) can prevent upper respiratory tract infections in children (7, 8). Other studies have demonstrated that gargling with hypertonic saline is protective against chronic inflammation in the nasal cavity (such as rhinitis and sinusitis) (9). Casale et al. (10) advocated saline nasal irrigation and oral rinse with antimicrobial agents to reduce the risk of COVID-19 infection. However, there are few clinical studies on nasal irrigation and oral rinse for the treatment of COVID-19.

Recent studies have shown that coronaviruses are unstable under alkaline conditions. Coronaviruses are rapidly and irreversibly inactivated by brief treatment at pH 8.0 and 37°C with a half-life of ~30 min (11). In addition, under alkaline conditions (pH > 8), the viral load inside host cells is greatly reduced (12). All these evidences indicate that the infectivity of coronaviruses is pH-dependent (13). Sodium bicarbonate is a widely used alkaline reagent. Nasal irrigation with sodium bicarbonate has been used for the treatment of rhinitis and nasopharyngeal diseases (14). The Omicron variant mainly infects the upper respiratory tract (15), and the nose and mouth are main entry portals for SARS-CoV-2 to enter a human body (16). Thus, we proposed that nasal irrigation and oral rinse with sodium bicarbonate solution might be effective in the treatment of COVID-19. We conducted this pilot study to observe the effect of nasal irrigation and oral rinse with 5% sodium bicarbonate solution on virus clearance for COVID-19 patients.

## 2. Materials and methods

COVID-19 patients were recruited according to the following criteria.

### 2.1. Inclusion criteria

- All admitted patients with age more than 18 years.
- COVID-19 patients with mild or moderate symptoms.
- Positive RT-PCR result at the time of admission.

### 2.2. Exclusion criteria

- Refusal to participate.
- Any severe cases of COVID-19.
- Patients with a history of nasal surgery, chronic sinusitis or drug intervention through nasal cavity.
- Women in the period of pregnancy or lactation.
- Patients with immune deficiency (such as patients with malignant tumors, organ or bone marrow transplants, AIDS, and those taking immunosuppressive drugs within 3 months).
- Other patients that were considered unsuitable to participate in this study.

### 2.3. Study design

This was a randomized, open-label, single-center pilot study. Patients were observed for 4–5 days after hospitalization to exclude any severe cases of COVID-19, and then randomly divided into two group, i.e., the experimental group and the control group. The experimental group received regular care plus nasal irrigation and oral rinse with 5% sodium bicarbonate solution, while the control group received regular care only. Regular care refers to the treatment according to the “Diagnosis and Treatment Protocol for COVID-19 (Trial Version 9).” Oral rinse with sodium bicarbonate solution was conducted three times every day. Nasal irrigation was conducted two times in total. The solution entered through one nostril and exited from the other one, and the order of nostrils was changed on the second day. 30–40 mL of solution was used every time and the irrigation was performed for at least 30 s. Nasopharyngeal and oropharyngeal swab samples were collected from patients every day for RT-PCR tests. The specimen was considered positive if Ct (cycle threshold) values for both E and RdRP genes were smaller than 40, and the specimen was negative when no Ct value was obtained. If the RT-PCR results were negative, the patient would receive a second test after 24 h. The presence of two negative RT-PCR results, at ≥24-h intervals, was defined as viral clearance (17). Otherwise, the treatment was continued. If the RT-PCR results were positive, the patient should be observed to determine whether or not the situation got worse. If yes, the clinical trial was stopped immediately and the patient received further care. If not, the clinical trial was continued. This pilot study received approval from the Institutional Review Board of RuiJin Hospital LuWan Branch.

### 2.4. Data analysis

Statistical analysis was performed using the IBM SPSS Statistics (version 19). Categorical data were used for expressing frequency and percentage, and χ^2^-test was used for comparison between groups. For expressing mean ± standard deviation, continuous data were applied, and independent *t*-test was used for comparison between groups. A *p*-value of < 0.05 was considered as statistically significant.

## 3. Results and discussion

**Figure 1:**
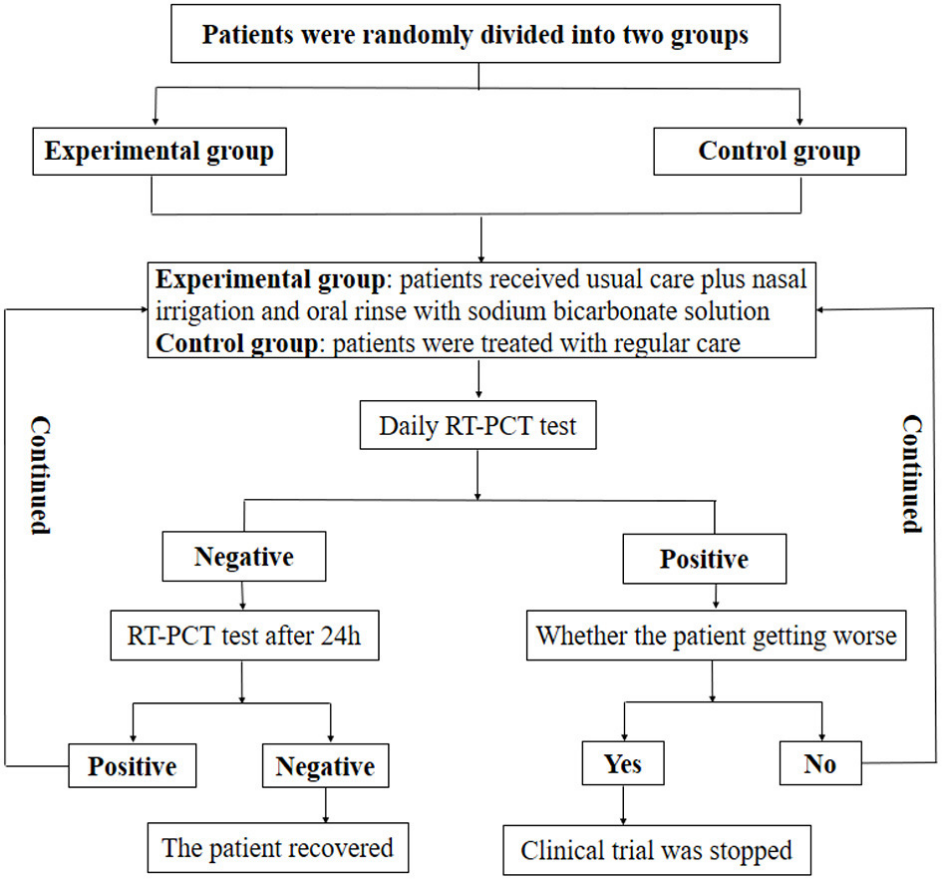
Flow chart of the clinical trial.

A total of 55 patients (26 males and 29 females) were included in our study. These patients were hospitalized because of positive RT-PCR results. The patients were randomly divided into two groups, 32 cases in the control group and 23 cases in the experimental group. The minimum and maximum ages were 35 and 100 years old, respectively. Patients were treated according to the procedure shown in Figure 1.

**Figure 2:**
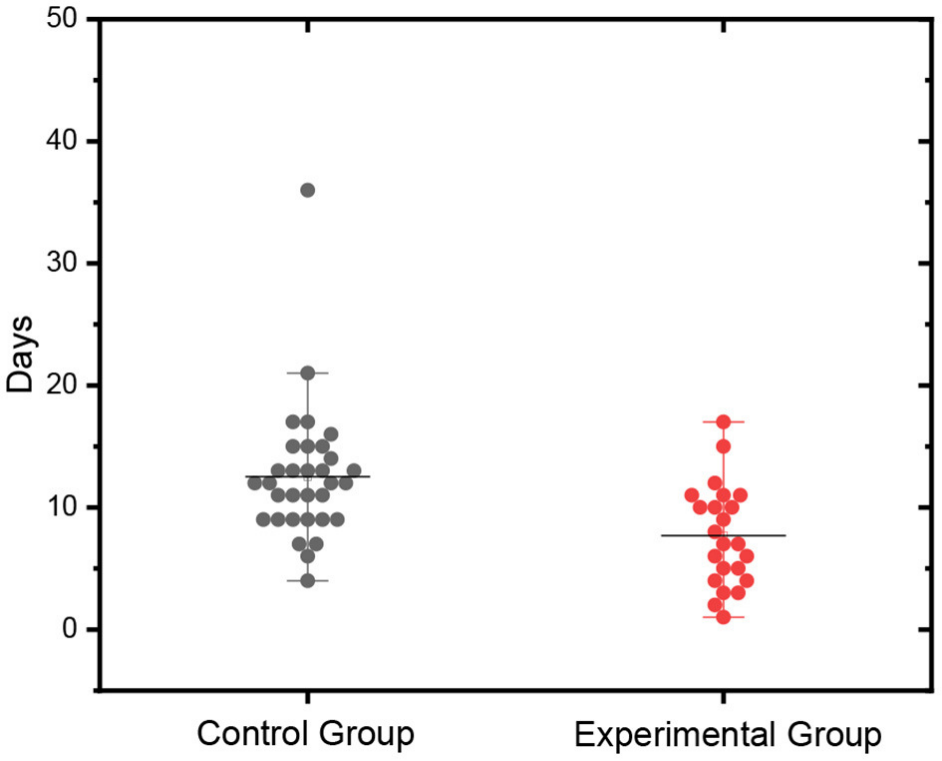
Scatter plots of hospitalization time.

Several important factors such as gender, age, and health status were studied. The mean ± SD of these factors are shown in Table 1. When the two groups were compared, *p*-values were all >0.05 for these factors, indicating that there was no significant difference between the experimental and control groups. Patients in the experimental group received regular care plus nasal irrigation and oral rinse with 5% sodium bicarbonate solution. They became negative in a very short time, and the average negative conversion time was 1.63 ± 1.25 days, with a total hospital stay of ~7.7 days. In contrast, the average hospitalization time for patients in the control group was 12.53 days, which was consistent with previous studies (18) and was significantly longer than that in the experimental group (*p*-value < 0.01). The scatter plots of hospitalization time are shown in Figure 2. The hospitalization time for patients in the experimental group was generally shorter than that in the control group. These results demonstrated that nasal irrigation and oral rinse with 5% sodium bicarbonate solution was effective in virus clearance for COVID-19 patients.

**Table 1:** Statistical analysis of difference between the two groups.

	**Experimental group (*n* = 23)**	**Control group (*n* = 32)**	**χ^2^-value**	***t*-value**	***p-*value**
Gender (male)	10 (43.48%)	16 (50.00%)	0.303		>0.05
Age	67.96 ± 11.22	65.91 ± 15.78		0.533	>0.05
Hypertension	11 (47.83%)	12 (37.50%)	0.311		>0.05
Coronary heart disease	4 (17.39%)	6 (18.75%)	0.508		>0.05
Diabetes	7 (30.43%)	5 (15.63%)	0.457		>0.05
Cerebrovascular disease	2 (8.70%)	1 (3.13%)	1.603		>0.05
Chronic respiratory disease	1 (4.35%)	0 (0.00%)	10.277		>0.05
Hospitalization time (days)	7.70 ± 4.15	12.53 ± 5.56		3.518	< 0.01

We proposed three possible reasons for this: (1) the main infection area of coronavirus is the upper respiratory tract. Most coronaviruses were washed away during nasal irrigation and oral rinse (19), resulting in a lower viral load. (2) Coronaviruses are unstable under alkaline conditions. The coronaviruses remaining in the nasopharynx and oropharynx were not stable due to the presence of sodium bicarbonate, which can be easily eliminated by the human immune system (11). (3) The entry of coronaviruses into host cells is pH-dependent (13). The number of coronaviruses entering the host cells decreased since the host cells were under alkaline conditions. All these factors contribute to the elimination of viruses. Thus, patients in the experimental group become negative in a shorter time. Although Kumar et. al. (20) reported that gargling with 7.5% sodium bicarbonate was ineffective in achieving early SARS-CoV-2 viral clearance for patients with mild COVID-19. This might be because the viral load in the nasal cavity is much higher than that in the mouth (21), and nasal irrigation is more effective in virus clearance than oral rinse.

Nasal irrigation and oral rinse is very simple, which can be operated by the patient. In addition, sodium bicarbonate is a cheap and widely used alkaline reagent. COVID-19 patients can rinse nose and mouth with sodium bicarbonate solution to clear coronaviruses even at home, which is significant for areas lack of medical resources. We believe this method is not only effective for COVID-19, it might be useful for some other diseases such as influenza.

## 4. Conclusions

The results of this pilot study demonstrated that nasal irrigation and oral rinse with 5% sodium bicarbonate solution was effective in reducing the viral load for COVID-19 patients. The sample size of this study was small. Some patients may already have symptoms before hospitalization, which might affect the total hospitalization time. Further studies with larger population are required to ascertain the benefits of nasal irrigation and oral rinse with sodium bicarbonate solution. Although this is only a preliminary study, the results are very promising. Nasal irrigation and oral rinse is simple and inexpensive. More importantly, this method can be performed at home, which might be an effective way to stop the spread of SARS-CoV-2.

## Data availability statement

The original contributions presented in the study are included in the article/supplementary material, further inquiries can be directed to the corresponding author.

## Ethics statement

The studies involving human participants were reviewed and approved by Institutional Review Board of RuiJin Hospital LuWan Branch. The patients/participants provided their written informed consent to participate in this study.

## Author contributions

TW, YZ, RZ, YM, JY, YL, QC, XL, HW, SH, CZ, and BT: methodology, investigation, and data curation. XW: writing—original draft, review and editing, conceptualization, supervision, and funding acquisition. All authors contributed to the article and approved the submitted version.
